# Predictive value of nodal metastases on local recurrence in the management of differentiated thyroid cancer. Retrospective clinical study

**DOI:** 10.1186/1471-2482-13-S2-S3

**Published:** 2013-10-08

**Authors:** Giovanni Conzo, Giovanni Docimo, Daniela Pasquali, Claudio Mauriello, Claudio Gambardella, Daniela Esposito, Ernesto Tartaglia, Cristina Della Pietra, Salvatore Napolitano, Antonia Rizzuto, Luigi Santini

**Affiliations:** 1 Department of Anesthesiologic, Surgical and Emergency Sciences Second University of Naples - Italy Via Pansini 5, 80131 Naples - Italy; 2 Department of medical and Surgical Sciences University of Magna Graecia Catanzaro - Italy

**Keywords:** Total thyroidectomy, Papillary thyroid cancer, Lymph node recurrence, Lymph node neck dissection, Radio active iodine ablation

## Abstract

**Background:**

The significance of nodal metastases, very common in papillary thyroid cancer, and the role of lymph node dissection in the neoplasm management, are still controversial. The impact of lymph node involvement on local recurrence and long-term survival remains subject of active research. With the aim to better analyze the predictive value of lymph node involvement on recurrence and survival, we investigated the clinico-pathological patterns of local relapse following total thyroidectomy associated with lymph node dissection, for clinical nodal metastases papillary thyroid cancer, in order to identify the preferred surgical treatment.

**Methods:**

Clinical records, between January 2000 and December 2006, of 69 patients undergoing total thyroidectomy associated with selective lymph node dissection for clinical nodal metastases papillary thyroid cancer, were retrospectively evaluated. Radioiodine ablation, followed by Thyroid Stimulating Hormone suppression therapy was recommended in every case. In patients with loco regional lymph nodal recurrence, a repeated lymph node dissection was carried out. The data were compared with those following total thyroidectomy not associated with lymph node dissection in 210 papillary thyroid cancer patients without lymph node involvement, at preoperative ultrasonography and intra operative inspection.

**Results:**

Incidence of permanent hypoparathyroidism (iPTH < 10 pg/ml) and permanent monolateral vocal fold paralysis were respectively 1.4 % (1/69) and 1.4% (1/69), similar to those reported after total thyroidectomy "alone". The rate of loco regional recurrence, with positive cervical lymph nodes, following 8 year follow-up, was 34.7% (24/69), higher than that reported in patients without nodal metastases (4.2%). A repeated lymph node dissection was carried out without significant complications.

**Conclusions:**

Nodal metastases are a predictor of local recurrence, and a higher rate of lymph node involvement is expected after therapeutic lymph node dissection associated with total thyroidectomy. The prognostic significance of nodal metastases on long-term survival remains unclear, and more prospective randomized trials are requested to better evaluate the benefits of different therapeutic approaches.

## Introduction

Papillary and follicular variants are the most frequent differentiated thyroid neoplasms [1,2], followed by medullary cancer, often part of the MEN-2 syndrome [3-5], and anaplastic carcinoma, associated with a prognosis similar to that reported in sarcomatoid carcinoma of other districts [6]. In the last ten years, ultrasound (US) guided fine-needle cytology (FNC) has allowed a more precocious diagnosis, identifying a higher number of small papillary cancer (SPC) [7-11]. Papillary thyroid cancer (PTC) prognosis is excellent, reflecting, with a 5- and 10-year survival of 90 and 95% [12], the favorable nature of the neoplasm. Recurrence develops in about 20% of patients, requiring additional treatment, responsible of significant morbidity, and PTC-related deaths are still reported [13,14]. The risk of relapse, in negative nodes patients, ranges between 0 and 9%, while clinically and US positive node cases are associated with higher recurrence rates, 10-42%, and also "high volume" disease is another unfavorable factor [15]. Differently from data reported in the management of breast, colorectal, gastro-esophageal, pancreatic, genitourinary or pulmonary cancers, thyroid metastatic nodes are associated with a higher mortality risk only in selected cases [16]. Despite the notable progresses in oncology, the true significance of the lymph node involvement, so frequent in pediatric and geriatric patients, is unclear and object of active research. There is agreement about its impact on local recurrence [16] but, according to more recent data, nodal metastases may affect long-term survival only in high risk patients [17]. Also metastatic lymph node number and size may be of prognostic value [16], but unfortunately the extent of LD and its indications - routine vs therapeutic- are still matter of intensive research [15]. The analysis of the clinico- pathological patterns of local recurrence in patients undergone LD, associated with TT for N+ PTCs, and the predictive value of nodal metastasis on management outcomes were the main objectives of our retrospective study.

## Study design

Clinical records of PTC patients, undergoing TT associated with LD, between January 2000 and December 2006, for clinical metastatic nodes, identified at preoperative ultrasonography or intraoperative inspection, were analyzed. They were compared with those observed in a clinical series of 210 patients undergone TT without LD for N0 PTC. In every case, a preoperative diagnosis of PTC was obtained by US guided FNC. The preoperative work-up consisted of thyroid hormones, TSH, Tg and anti-Tg antibodies levels evaluation, and a high resolution ultrasonography of the neck. A pre- and postoperative fibrolaryngoscopy was performed in all patients. In 8 patients (11.5%) BRAF mutations were searched on tumor specimens. Tumor extent was evaluated according to the American Joint Committee on Cancer (AJCC) TNM Classification of Thyroid Cancer (7^th ^edition, 2010). Postoperative diagnosis of lymph node recurrence was detected by US guided FNC, and Tg washing of FNC aspirates, performed in cases of enlarged lymph node ≥ 1 cm. Patient demographics, postoperative complications, including neck hematoma requiring reoperation, transient or permanent hypoparathyroidism, transient or permanent vocal cord palsy, distant and loco regional recurrence, detected by postoperative surveillance, were recorded. In case of a iPTH level < 10 pg/ml (normal value = 10-65), hypoparathyroidism was determined, and it was considered permanent if persisting for more than 6 months and requiring medical therapy. Vocal fold palsy, confirmed by fibrolaryngoscopy, was considered permanent when lasting for more than 6 months. Qualitative data were expressed in percent, while quantitative data as means.

## Materials and methods

Tg and TSH was determined by Immunite immunoassay (Siemens Healthcare Diagnostics) with sensitivities of 0.2 ng/mL and 0.03 mIU/L, respectively. Anti-Tg antibodies were detected by Quanta Lite enzyme-linked immunosorbent assay (Inova Diagnostics), and we considered 40 UI/ml as cut-off value of Anti-Tg Abs (normal value = 0-60 UI/ml). Each surgeon participating to the study used a similar technique in performing TT, as well as central and lateral lymph node dissection. An ultrasonic scalpel, Harmonic Ace ^® ^( Ethicon Endosurgery) was utilized in 9 cases (13%), and in selected cases hemostasis was optimized by means of Floseal ^® ^Hemostatic Matrix (Baxter Zurich- Switzerland). Routinely, recurrent laryngeal nerves were identified and exposed, as well as parathyroid glands were identified and preserved. In case of suspected devascularized or incidentally removed parathyroid glands, a muscular autoimplantation followed. Serum calcium and intact parathormone levels were assayed on the first postoperative day, and subsequently on a clinical basis evaluation. In case of loco regional recurrence, a selective central or ipsilateral (VI, III, IV,V) LD was performed according to the American Academy of Otolaryngology - Head and Neck Surgery [17]. After surgery, all patients underwent adjuvant RAI ablation (1850-3700 MBq-^131^I ). Apart from lymph node involvement, indications for postoperative ^131^I treatment are a tumor > 1.0 cm, extra-capsular thyroid invasion or loco regional extension, an unfavorable histological subtype (follicular, diffuse sclerosing or tall cells papillary cancer), multifocal disease, BRAF positive tumor specimens. To obtain adequate levels of endogenous TSH (>30 mU/ml), that are associated with an increased radioiodine uptake, patients stopped L-T4 replacement 3-4 weeks before radioiodine treatment; when L-T4 withdrawal was not indicated, TSH stimulation was achieved with Recombinant Human Thyrotropin (rhTSH) (Thyrogen ^® ^- Genzyme corporation). Post-therapy whole-body scan was performed 4-7 days after RAI treatment. Neck ultrasonography and monitoring of serum Tg and Tg-antibodies levels, during suppressive l-tiroxine treatment, were carried out every 6 months. A serum Tg level ≤1 ng/ml was considered as undetectable. Surveillance for possible recurrence in patients considered disease-free was achieved by Tg detection after rhTSH stimulation (American Thyroid Association Guidelines 2009) and neck ultrasound. Diagnosis of disease recurrence in the cervical lymph nodes was based on US-guided FNC, Tg washing of FNC aspirates and serum Tg levels monitoring.

## Results

Sixty-nine PTC patients, 45 women and 24 men (F/M ratio = 1.8/1), with a 34-year mean age (19-70) were submitted to TT associated to selective LD (Table 1). In 5/69 patients (7.24%), parathyroid tissue was implanted in the strap muscles, and in 9/69 cases (13%) parathyroid tissue was identified in the final pathology analysis. Incidence of surgical complications is reported in Table 2. The mean primary tumor size was respectively 1.36 cm (0.2-5) in TT and LD group and 1.4 cm (0.7-2.4) in TT patients, and a microcarcinoma (≤1 cm) was diagnosed in 6 patients (8.69 %) irrespective to 29% of TT group. Histotype was classic in 60 patients (86.9%), follicular variant in 9 (13%); 12 patients (17.39%) had multifocal tumors - 9/12 classic variant, 3/12 follicular variant vs 15.2% of TT group. Nine out of 69 patients (13%) had a loco regional infiltration (T3), vs 11.4% of TT patients, by classic variant tumor in 7 cases and by follicular variant tumor in 2 cases. A BRAF mutation was discovered in 1 out of 10 tested patients (10%). Patients pTNM Stage and pathological data are shown in Table 1. No patient developed distant recurrence during a 8-year mean follow-up (6-13).

**Table 1:** Demographic and pathological data of 69 N+ PTC patients

		%
Patients	Male	34.7
	Female	65.2
	Mean age	34 (19-70 years)
Histology	Classic	86.9
	Follicular-variant	13
Tumor	Mean size	1.36 (0.2-5 cm)
	Unique	82.61
	Multifocal	17.39
	Microcarcinoma	8.69
	Locoregional infiltration	13
pTNM stage	I	55
	II	21.7
	III	23.1
Lymph node recurrence		34,7

**Table 2:** Total Thyroidectomy and LD: complications

	%	N. Pts
Temporary Hypoparathyroidism	11.59	8/69
Permanent Hypoparathyroidism	1.4	1/69
Parathyroid tissue in the specimen	13	9/69
Temporary unilateral VCP	4.34	3/69
Permanent unilateral VCP	1.4	1/69
Bilateral VCP	0	0/69

VCP= vocal cord palsy

### Lymph nodal recurrence

Following TT associated with LD and RAI ablation, 24/69 cases (34.7%) of nodal recurrence - 6 central recurrence (VI) and 18 ipsilateral recurrence (III-IV) - were observed, representing a rate higher than reported in patients without preoperative clinical nodal metastases (4.2%). Demographic characteristics were the following: 14 males - median age 33.25 years (27-38) - and 10 females - median age 27.25 years (19-30) with a M/F ratio = 1.4/1. The median elapsed time between TT and lymph node recurrence was 44 months (4-85). Twenty-one patients had classic variant, 3 had follicular variant PTC; median Tg value was 2.74 ng/ml (0.1-13.1 ng/ml) ( Tg>2 ng/ml in 5 patients and <2 ng/ml in 19 patients) and Tg-antibodies value was > 40 UI/ml in 10 patients and <40 UI/ml in 14 (33.5-722 UI/ml). In every case, a repeated central and ipsilateral lymph node dissection (VI,III,IV,V) was performed, followed by another session of metabolic radiotherapy. Significant perioperative complications were not observed, and the mean number of removed lymph nodes, as documented by pathologists, was 16.5 (2-32). Metastases were reported in up to 2.8 (1-5) collected lymph nodes.

## Discussion

According to our data, a higher loco regional recurrence rate with a statistically significant difference, 34.7 vs 4.2%, following TT associated with therapeutic selective LD, was observed irrespective to nodal relapse rate reported after TT, in absence of lymphatic involvement. Metastatic lymph node was a predictive factor, favoring recurrent disease after 8-year mean follow-up. In absence of suspected enlarged lymph node, identified by preoperative ultrasonography and intraoperative inspection, patients undergoing TT without routine central lymph node dissection (RCLD) have a low risk of lymph nodal recurrence. In the treatment of low-risk PTC patients, TT and RAI ablation allow favorable long-term results; regarding recurrence and survival, routine LD is not indicated and needs to be more intensively investigated. About recurrence, male gender (a male/female ratio of 1.4/1) and age <50 years were the main clinical features, while the follicular variant was frequently associated with lymph node relapse. Primary tumor mean size was <2 cm, with the lateral compartment mostly affected, and a median elapsed time between TT, associated with LD, and lymph node recurrence of 44 months (4-85). The prognostic impact of the postoperative RAI treatment, administered in all of the LD patients, as well as in most TT "alone" cases, must be considered similar in the two groups. The small number of examined N+ PTC cases, the absence of a control group of patients undergoing RCLD, and the inability to exactly distinguish between recurrent and persistent disease, were the main limitations of this retrospective analysis. PTC multifocal nature, the effectiveness of RAI treatment and the monitoring of serum thyroglobulin (Tg) levels during follow-up are evocated in favor of TT and of the TSH suppressive therapy. On the contrary, the role of the LD, regarding its indication- routine or therapeutic-, extension - ipsi or contralateral- and outcomes, in terms of recurrence and survival, and postoperative RAI ablation are subject of active research. According to the recent American Thyroid Association (ATA) guide lines, RCLD is indicated in high risk patients with advanced primary tumors [17], but prospective randomized trials are needed to evaluate its benefits [19]. Relapse prevention, the high risk of positive lymph nodes and the lower morbidity rate of the first operation are evocated in favor of RCLD [20], often associated with an higher rate of complications, without demonstrable benefits in terms of long-term survival [21,22]. The high rate of micro and clinical nodal metastases is in contrast with the low incidence of clinical recurrence following TT without LD, and remains an "obscure" issue in oncology. According to recent studies, nodal metastases may affect recurrence and survival rates especially in older patients [13]. Multifocal primary tumor, infiltration of thyroid capsule, patient age (pediatric or geriatric population), tumor size greater than 3 cm, several oncogenes (p53, BRAF), nodal metastases (number and size), as demonstrated in our series, are considered the main risk factors for local recurrence [16,23]. Especially in male patients > 45 years, with aggressive histotypes, capsular or locoregional infiltration, incomplete tumor resection, BRAF positivity, a higher risk of loco regional and distant recurrence is reported, while in the management of low-risk patients, without suspected lymph node, TT, associated with a low morbidity, similar to that reported in parathyroid surgery [24-29], remains the operation of choice, as in most thyroid diseases [30-35]. RCLD, failed to demonstrate beneficial effects on recurrence and long-term survival, increasing the rates of permanent hypoparathyroidism and of unintentional permanent recurrent laryngeal nerve injury [15,20,36-38]. At the same time literature data demonstrated that reoperation (lymph node dissection) is not associated with a higher morbidity [39,40], and we believe that, in absence of suspicious enlarged lymph node, routine central or lateral LD is not routinely indicated.

## Conclusions

Our study supports the following data. Locoregional recurrence rate, mostly of the lateral compartment, frequently observed 2 years after surgery in young male patients, was higher in N+ patients undergone therapeutic selective LD. A lower relapse rate was observed in N0 PTC following TT alone, confirming the predictive value of risk of lymph node involvement. PTC classic variant, in every case less than 2 cm was the most frequent observed hystotype. In the treatment of low-risk PTC patients, routine LD is not indicated. Considering the controversial literature data, further prospective randomized studies are needed in the attempt to clarify the predictive value of lymph node involvement on long-term survival.

## Abbreviations

PTC: papillary thyroid cancer; LD: lymph node dissection; TT: total thyroidectomy; N+: clinical nodal metastases; RAI: Radioiodine; TSH: Thyroid Stimulating Hormone; US: ultrasound; FNC: guided fine-needle cytology; SPC: small papillary cancer; PTC: Papillary thyroid cancer; rhTSH: Recombinant Human Thyrotropin; RCLD: routine central lymph node dissection; Tg: serum thyroglobulin; ATA: American Thyroid Association

## Competing interests

The authors declare that they have no competing interests.

## Authors' contributions

GC: conception, design, and execution of the study; critical revision; analysis and interpretation of data; drafting and editing of the manuscript; given final approval of the version to be published.

GD: conception, design, and execution of the study; analysis and interpretation of data; drafting and editing of the manuscript.

DP: conception, design, and execution of the study; analysis and interpretation of data; drafting and editing of the manuscript.

DE: conception, design, and execution of the study

ET: conception, design, and execution of the study

CDP: conception, design, and execution of the study; analysis and interpretation of data.

SN: conception, design, and execution of the study

AR: conception, design, and execution of the study

LS: conception and design of the study, given final approval of the version to be published.

## Authors' information

GC: Assistant Professor of Surgery at Second University of Naples

GD: Associate Professor of Surgery at Second University of Naples

DP: Assistant Professor of Endocrinology at Second University of Naples

CM: Surgical fellow at Second University of Naples

CG: Surgical fellow at Second University of Naples

DE: Surgical fellow at Second University of Naples

ET: Surgical fellow at Second University of Naples

CDP: Surgical fellow at Second University of Naples

SN: Surgical fellow at Second University of Naples

AR: Surgical fellow at University of Catanzaro

LS: Full Professor of Surgery at Second University of Naples

## Declaration statement

Publication of this article was funded by personal funds.

This article has been published as part of *BMC Surgery *Volume 13 Supplement 2, 2013: Proceedings from the 26th National Congress of the Italian Society of Geriatric Surgery. The full contents of the supplement are available online at http://www.biomedcentral.com/bmcsurg/supplements/13/S2
